# Waterpipe tobacco smoking: an urgent public health concern among adolescents and young adults

**DOI:** 10.1097/MS9.0000000000004381

**Published:** 2025-11-24

**Authors:** Saniya Devi, Sanhia Maheshwari, Najeeb Ahmed, Muddassir Khalid

**Affiliations:** a Department of Medicine, Jinnah Sindh Medical University, Karachi, Pakistan; b Department of Medicine, Liaquat University of Medical and Health Sciences, Jamshoro, Pakistan; c Department of Medicine, Nishtar Medical University, Multan, Pakistan

Dear Editor,

Waterpipe tobacco smoking is increasingly common, especially among adolescents and young adults, driven by peer influence, social acceptance, and misconceptions about its safety^[1]^. A recent study discovered that approximately 25% of students have ever smoked a waterpipe, frequently as a result of a lack of knowledge regarding the health effects and hazardous constituents of the substance. The rates of waterpipe use were four times higher among students with limited awareness, and the highest rates were observed among senior students^[2]^.

Smoke from waterpipes contains a higher concentration of nicotine than cigarettes. Users are exposed to elevated concentrations of toxic flavoring agents, including pulmonary irritants such as cinnamaldehyde^[3]^. Oxidative stress and inflammation can result in endothelial cell injury. This condition is comparable to that caused by cigarettes, even after a single waterpipe session^[4]^. Similar to other tobacco products, nicotine dependence can result from prolonged use, indicating its potential for addiction^[5]^. Research suggests that healthy adult waterpipe consumers experience substantial impairments in memory, attention, and alertness, which are closely linked to elevated levels of anxiety and depressive symptoms^[6]^. Additionally, there is a correlation between respiratory disease, cardiovascular disease, esophageal and lung malignancies, periodontal disease, and obstetric complications, including low birth weight^[7]^. The transmission of communicable diseases has also been linked to the use of communal water pipes. The World Health Organization has issued a warning regarding the transmission of herpes, *H. pylori*, hepatitis, and fungal infections. Pulmonary tuberculosis was associated with the regular smoking of communal waterpipes in a case report that was indexed in PubMed^[8]^. Additionally, prominent brands, including those that manufacture e-cigarettes and hookahs, are utilizing social media to market and promote their products. They present tobacco use as a socially permissible behavior among young adults and normalize it^[9]^.

Consequently, it is imperative to implement awareness campaigns and rigorous regulations regarding waterpipe marketing, particularly on social media, to dispel misconceptions and reduce the initiation of waterpipe use among adolescents and young adults. Furthermore, we support the implementation of proactive interventions to reduce marketing strategies and prevent waterpipe smoking from being portrayed as a safe, socially acceptable alternative to cigarette smoking, as well as increased public awareness and additional research on the detrimental effects of waterpipe smoking.

TITAN Guidelines: This manuscript is in compliant to TITAN Guidelines, 2025, declaring no use of AI^[10]^.

## Data Availability

Data available on request from the authors.
